# Climate change, transgenic corn adoption and field-evolved resistance in corn earworm

**DOI:** 10.1098/rsos.170210

**Published:** 2017-06-07

**Authors:** P. Dilip Venugopal, Galen P. Dively

**Affiliations:** 1 Science and Technology Policy Fellow, American Association for the Advancement of Science, Hosted by Transportation and Climate Division, Office of Transportation and Air Quality, United States Environmental Protection Agency, 1200 Pennsylvania Avenue NW, Washington, DC 20004, USA; 2 Department of Entomology, University of Maryland, 4112 Plant Sciences Building, 4291 Fieldhouse Dr, College Park, MD 20742, USA

**Keywords:** climate change and phenology, temperature anomaly, transgenic crops, *Helicoverpa zea*, crop–pest interactions, insect resistance

## Abstract

Increased temperature anomaly during the twenty-first century coincides with the proliferation of transgenic crops containing the bacterium *Bacillus thuringiensis* (Berliner) (Bt) to express insecticidal Cry proteins. Increasing temperatures profoundly affect insect life histories and agricultural pest management. However, the implications of climate change on Bt crop–pest interactions and insect resistance to Bt crops remains unexamined. We analysed the relationship of temperature anomaly and Bt adoption with field-evolved resistance to Cry1Ab Bt sweet corn in a major pest, *Helicoverpa zea* (Boddie). Increased Bt adoption during 1996–2016 suppressed *H. zea* populations, but increased temperature anomaly buffers population reduction. Temperature anomaly and its interaction with elevated selection pressure from high Bt acreage probably accelerated the Bt-resistance development. *Helicoverpa zea* damage to corn ears, kernel area consumed, mean instars and proportion of late instars in Bt varieties increased with Bt adoption and temperature anomaly, through additive or interactive effects. Risk of Bt-resistant *H. zea* spreading is high given extensive Bt adoption, and the expected increase in overwintering and migration. Our study highlights the challenges posed by climate change for Bt biotechnology-based agricultural pest management, and the need to incorporate evolutionary processes affected by climate change into Bt-resistance management programmes.

## 1. Introduction

Climatic change, as indicated by the highest global average temperatures on record, impacts agricultural productivity globally. Sixteen of the 17 warmest years recorded since 1880 occurred in the twenty-first century, and land surface temperature anomaly (difference as compared to twentieth century) during 2016 reached 1.43°C [1]. With the increasing average temperatures, agricultural yield losses and management costs are expected to rise due to the changes in geographical range and infestation intensity of crop pests [2,3]. Pests exacerbate agricultural costs of climate change; thus, incorporating their effects on agricultural productivity can aid in developing robust climate change mitigation strategies [2,4]. However, crop–pest interactions and biological control effects are complex and poorly understood in the context of climate change. Direct climate impacts on pests (e.g. life-history traits) are better understood than the indirect effects (such as crop–pest interactions and biological control) resulting in a gap between the science of predicting changes and management options [4–6].

Insects are major economic pests, and increasing temperature anomalies and warming have profound influences on insect biology and agricultural pest management. Rising temperatures accelerate insect phenology [7], and could exacerbate agricultural problems from migratory pests [8]. Polyphagous, migratory insect pest species can benefit from warmer temperatures [7–9], although climate change effects on insects in general are varied. Migratory agricultural insect pest responses to warmer temperatures include extension of geographical and overwintering range, increased overwintering survival, accelerated population growth, increased number of generations, increased feeding injury and earlier colonization of crops. These responses may alter the crop–pest synchrony, interactions among species, and pest management strategies [5,7–13].

Currently, a key insect pest management strategy is the genetic engineering of crops through agricultural biotechnology. The period of increased temperature anomaly in the twenty-first century coincides with the proliferation of crops engineered with genes from the bacterium *Bacillus thuringiensis* (Berliner) (Bt) to express insecticidal toxins. Bt crops are safe for human health and provide many benefits through reduced pest populations and pesticide usage, and increased profits [14–17]. Bt crops are adopted extensively with global plantings across 28 countries reaching about 178 million hectare in 2015, from 1.1 million hectare in 1996 [18]. However, the implications of altered pest insect biology and phenology during warmer climate on transgenic crop–pest interactions are complex and largely unexamined. In this study, we address the role of increasing temperature anomaly and its interaction with agricultural biotechnology (Bt crops) to understand some of the indirect impacts of climate change: crop–pest interaction, efficacy of Bt corn for insect pest management and Bt resistance in insects.

Agricultural biotechnology, through molecular breeding and genetic engineering, could significantly help to mitigate the effects of climate change on agricultural production [19,20]. To use agricultural biotechnology for mitigating climate change impacts, we need to understand its risks and limitations versus benefits. The regional adoption of Bt maize (corn; *Zea mays* L.) and Bt cotton (*Gossypium* sp*.* L*.*) provides economic benefits by suppressing pest populations and damage from lepidopteran pests [14,17,21–23]. Bt adoption, however, exacerbates selection pressure and risks of resistance evolution because the ‘high dosage’ of Bt toxin, a requirement for resistance management [24], is not expressed for all targeted pests. With widespread use of Bt crops, insect resistance is the major threat to the sustainability of the Bt transgenic technology [25]. Resistance to the insecticidal toxins are on the rise with five major insect pest species developing field-evolved resistance (*sensu* Tabashnik *et al.* [26]) to Bt crops worldwide [25,27].

While the role of biotechnology regulatory policies and insect resistance management strategies for the sustainability of Bt technology is well documented [28], Bt crop–pest interactions and Bt crop efficacy in the context of climate change has received scant attention. Evolutionary adaptation to counter ecological stress during climate change may be rapid [29], and understanding its relevance for Bt biotechnology will help pest management. Climate change, through increased voltinism [5,7,30], may buffer the population reduction from Bt crops especially for polyphagous crop pests targeted by Bt technology. Environmental factors such as temperature, salinity, water logging or drought conditions affect the expression of Bt toxin proteins in transgenic plants, and their efficacy [31–35]. Coupled with accelerated phenology and increasing ranges (overwintering and distributional) of target pests [11,36], climate change effects pose increased risks for resistance development and sustainability of the Bt technology. Knowledge of the effects of environmental factors on the efficacy of transgenic plants and pest-resistance development is essential for sustainability of Bt technology, and to effectively use it for pest management in the context of climate change.

One of the targets of the Bt crops is the polyphagous corn earworm, *Helicoverpa zea* (Boddie), a key pest of many agricultural crops, including sweet corn, field corn, cotton, tomato, *Solanum lycopersicum* L., and grain sorghums, *Sorghum bicolor* (L.). In North America, *H. zea* is a long-distant migrant [37] distributed throughout most of eastern and mid-western USA and southern Canada. As pupae that diapause in the soil, it overwinters in areas south of 40° N [38]. However, its overwintering and distribution ranges are predicted to expand due to warmer temperatures [11], leading to changes in arrival and intensity of infestations in northern latitudes [39].

For all Bt corn expressing Cry toxins, the high dose requirement for resistance management is not achieved for *H. zea* which is more tolerant to the Bt toxins. Owing to this ‘moderate dose’ expression, the risk of evolution of resistance increases in areas where Bt field corn is widely adopted, and *H. zea* overwinters successfully [40]. Recently, Dively *et al.* [25] described and characterized the field-evolved resistance in *H. zea* to multiple Cry toxins, including pyramided traits expressed in transgenic sweet corn. During 1996–2016, the rate of increase for the indicators of resistance was significantly different between Bt and non-Bt hybrids with steeper increases for Bt than non-Bt hybrids (see fig. 1*a–d* in Dively *et al.* [25]), clearly demonstrating the temporal trend of resistance accumulation. Many sweet corn farmers in Maryland, USA either have stopped growing these Bt hybrids or are applying more insecticide sprays to compensate for the reduced control efficacy. With resistant populations in Maryland, which historically represented northern range of overwintering [41], *H. zea* resistance to Bt corn may spread and increase.

Simulations predict that the regional deployment of Bt crops affects average gene frequencies, and therefore incomplete technology adoption can slow resistance evolution [42,43]. Conversely, extensive adoption of Bt technology increases selection pressure for *H. zea* resistance evolution. Contrastingly, models also predict that resistance could evolve rapidly in other *Helicoverpa* sp. when Bt crops are rare in the landscape, rather than common [44]. However, field evaluations of these simulation models quantifying the relationship between *H. zea* resistance to Cry proteins and Bt crop adoption in the landscape are not available. Such information is necessary to predict vulnerability of crops to increased damage due to the spread of Bt resistant *H. zea* under climate change scenarios. In this study, we use two decades of data on indicators of resistance to Bt sweet corn and examine the role of temperature anomaly and Bt adoption on field-evolved resistance in *H. zea* to Cry toxins.

## 2. Material and methods

### 2.1. Indicators of field-evolved resistance

We used the data provided by Dively *et al.* [25,45] that indicate field-evolved resistance of *H. zea* to Bt Cry proteins (measures of control efficacy and changes in susceptibility). Dively *et al.* [25] established sentinel plots of Cry1Ab expressing Bt sweet corn hybrids paired with non-Bt isogenic hybrids, and evaluated *H. zea* infestations and abundances during 1996–2016 in Maryland, USA. We used percentage of ears damaged by *H. zea*, mean kernel area consumed, mean instars and proportion of late instars (4th–6th instars) in Bt hybrids (henceforth damage, consumption, instar and late instars, respectively) as indicators of field-evolved resistance to Cry1Ab in sweet corn. See Dively *et al.* [25] for details on field protocols and data collection procedures. We used the mean nightly captures of *H. zea* moths in black light traps during 1996–2014 (henceforth counts) as measure of population abundance.

### 2.2. Transgenic corn acreage and temperature anomaly data

As predictors of field-evolved resistance, we used Bt corn (including field corn) acreage in the agricultural crop districts of the study sites in Maryland (https://www.nass.usda.gov/Charts_and_Maps/Crops_County/boundary_maps/md.gif) as a measure of Bt adoption. The Bt events and Cry proteins expressed in field corn are also expressed in sweet corn (except for Cry1F). For e.g. Attribute (expressing Cry1Ab toxin, event Bt11) and Attribute II (expressing Cry1Ab and Vip3A, event MIR162) hybrids from Syngenta Seeds, and Performance Series (PS) hybrids (expressing the Cry1A.105 and Cry2Ab2 toxins, event MON89034) from Seminis Seeds. Therefore, Bt field corn acreage, which represented 81% of all corn acreage in the USA in 2015, is a good measure of the evolutionary selection pressure for resistance development in *H. zea* against Bt Cry proteins. Area under corn production differed among the study sites, representing a range of selection pressure for resistance development. Crops (particularly corn) are planted extensively around the sites on the Eastern Shore, while western Maryland sites include urban areas. We estimated the Bt corn acreage in the agricultural districts as the product of total area of corn planted each year [46] and the national average percentage of Bt corn for that year [47].

We compiled the temperature anomaly data for the respective climatic divisions (https://www.ncdc.noaa.gov/monitoring-references/maps/us-climate-divisions.php). Temperature anomalies, particularly warmer temperatures, during the growing season (spring to summer) are related to increased number of generations [7], earlier arrival of migratory insect pests and increased feeding and crop damage [8]. We downloaded data on temperature anomaly during the growing seasons (spring–summer; April–September) of the study period (1996–2016), for each agricultural district, from NOAA—National Climatic Data Center (http://www.ncdc.noaa.gov/cag/time-series).

### 2.3. Statistical analysis

Linear mixed effects models (LMMs) were used to analyse the influence of temperature anomaly and Bt acreage on *H. zea* abundance and on indicators of field-evolved resistance. The LMMs contained each of the indicators (counts, damage, consumption, instar and large instar) as response variables, site as a random effect accounting for repeated measurement, and the interaction, additive, or individual effect of (log) Bt acreage and temperature anomaly as fixed effects. We tested the interactive influence of Bt acreage and temperature anomaly prior to analysing additive or individual influences. Where applicable, the response variables were square root transformed to conform to linear model assumptions.

We determined the statistical significance of the fixed effects through Wald *F*-tests with Kenward–Roger approximation. Also, a likelihood-ratio based pseudo-*R*^2^ value [48] was calculated for each model as a measure of the proportion of total variance explained. We ensured model appropriateness through diagnostic plots of the models visualizing within-group residuals (standardized residuals versus fitted values, normal Q-Q plots, histograms of residuals) and estimated random effects (normal Q-Q plots and pairs-scatter plot matrix; see [49]; pp. 174–197). Package ‘lme4’ [50] was used to construct the LMMs, and pseudo-*R*^2^ values were calculated using package ‘MuMIn’ [51]. Estimated coefficients were extracted from the LMMs and plotted using ‘ggplot2’ [52] and ‘visreg’ [53], all in R program [54].

## 3. Results

**Figure 1.**
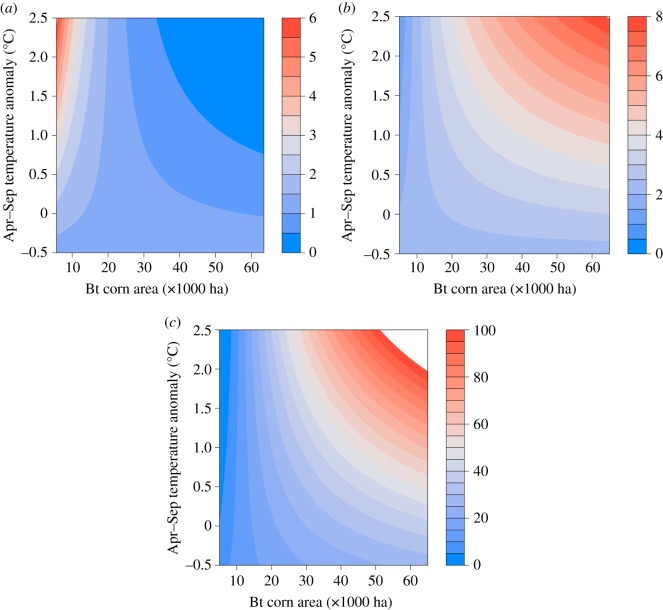
Interactive influences of temperature anomaly and acreage of Bt corn in the agricultural districts of Maryland, USA during 1996–2016 on: (*a*) *Helicoverpa zea* population abundance (Mean nightly *H. zea* captures); and indicators of field-evolved resistance to Bt corn expressed as (*b*) mean kernel area consumed (cm^2^) and (*c*) proportion of 4th–6th instars. We estimated Bt corn acreage as the product of total area of planted corn for each year in each agricultural district and the national average percentage of Bt corn for that year. We used temperature anomaly (compared to twentieth-century averages) during the growing season (April–September) of each year of the study period as a predictor. Contour plots depict the predictions from LMMs with each dependent variable scaled on the right axis, with lower values in blue and higher in red.

The interactive influences of temperature anomaly and log Bt acreage significantly influenced the abundance of *H. zea* over the past two decades (*F*_1,77_ = 35.6, *p* < 0.001, pseudo-*R*^2^ = 0.53; see table 1 for coefficients). Results show contrasting effects of the variables on abundance, with the effect of temperature anomaly decreasing with increasing Bt acreage. While the overall population trend was negative, abundance was highest at low Bt acreage and high-temperature anomaly (figure 1*a*).

**Table 1.** Summary of statistical analysis on the role of temperature anomaly and extent of Bt acreage in the agricultural districts on indicators of field-evolved resistance in *Helicoverpa zea* to Cry1Ab Bt expressing sweet corn hybrids during 1996–2016 in Maryland, USA.

response variable/data transformation	fixed effects	model parameters	coefficient estimate	s.e.
mean nightly *H. zea* captures square root	temperature anomaly + log (Bt acreage) + temperature anomaly: log (Bt acreage)	intercept	1.58	0.20
		temperature anomaly	1.05	0.16
		log (Bt acreage)	−0.15	0.04
		temperature anomaly: log (Bt acreage)	−0.34	0.06
damaged ears (%)	temperature anomaly + log (Bt acreage)	intercept	10.07	7.11
		temperature anomaly	16.32	3.52
		log (Bt acreage)	9.00	2.17
mean area consumed (cm^2^) square root	temperature anomaly + log (Bt acreage) + temperature anomaly: log (Bt acreage)	intercept	0.85	0.20
		temperature anomaly	−0.54	0.24
		log (Bt acreage)	0.12	0.06
		temperature anomaly: log (Bt acreage)	0.24	0.08
mean instar	log (Bt acreage)	intercept	2.37	0.23
		log (Bt acreage)	0.27	0.07
proportion of late instars (4th–6th) square root	temperature anomaly + log (Bt acreage) + temperature anomaly: log (Bt acreage)	intercept	−0.38	1.15
		temperature anomaly	−2.69	0.64
		log (Bt acreage)	1.52	0.20
		temperature anomaly: log (Bt acreage)	1.11	0.23

**Figure 2.**
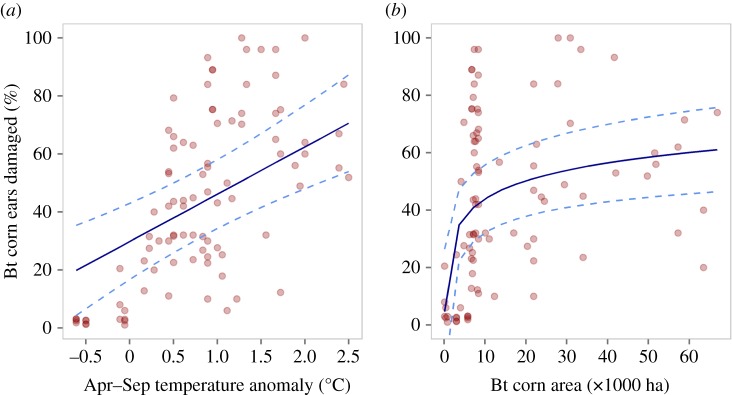
Additive influences of temperature anomaly (*a*) and acreage of Bt corn in the agricultural districts (*b*) during 1996–2016 on damage to Bt sweet corn (% ears damaged) by *Helicoverpa zea*, as indicator of field-evolved resistance to Bt corn. We estimated Bt corn acreage as the product of total area of planted corn for each year in each agricultural district of Maryland, USA and the national average percentage of Bt corn for that year. We used temperature anomaly (compared to twentieth-century averages) during the growing season (April–September) of each year of the study period as a predictor. Points represent the raw data, blue line represents the predictions from LMMs, and dotted lines denote the upper and lower confidence levels (95% CI).

**Figure 3.**
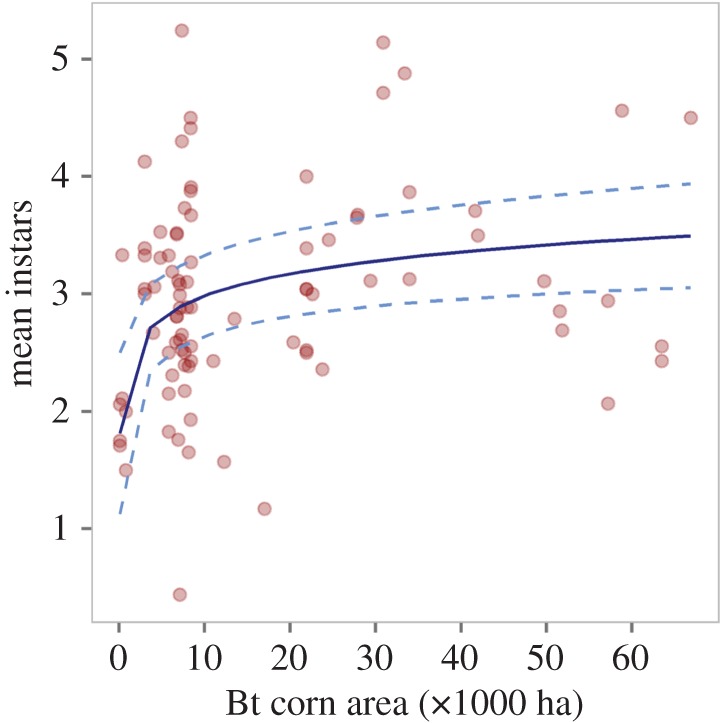
Influence of Bt corn acreage in the agricultural districts of Maryland, USA during 1996–2016 on mean instars of *Helicoverpa zea* on Bt sweet corn, as indicator of field-evolved resistance to Bt corn. We estimated Bt corn acreage as the product of total area of planted corn for each year in each agricultural district and the national average percentage of Bt corn for that year. Points represent the raw data, blue line represents the predictions from LMMs, and dotted lines denote the upper and lower confidence levels (95% CI).

For indicators of field-evolved resistance, damage was significantly positively associated with additive effects of temperature anomaly and log Bt acreage (temperature anomaly: *F*_1,89_ = 20.3, *p* < 0.001; Bt acreage: *F*_1,87_ = 15.9, *p* < 0.001, pseudo-*R*^2^ = 0.49). Damage to corn ears increased with temperature anomaly (figure 2*a*) and log Bt acreage (figure 2*b*; table 1 for coefficients). Kernel consumption of Bt hybrids was significantly influenced positively by the interactive effects of temperature anomaly and log Bt acreage (*F*_1,78_ = 7.06, *p* = 0.009, pseudo-*R*^2^ = 0.20; table 1 for coefficients). Kernel consumption was highest with interactive effects of high-temperature anomaly and log Bt acreage (figure 1*b*). The effect of temperature anomaly on kernel consumption increased with Bt acreage. Mean instar was significantly positively associated with Bt acreage (*F*_1,86_ = 13.7, *p* < 0.001, pseudo-*R*^2^ = 0.15; figure 3; table 1 for coefficients). The proportion of late instars was significantly positively associated with the interactive effects of temperature anomaly and log Bt acreage (*F*_1,147_ = 23.62, *p* < 0.001, pseudo-*R*^2^ = 0.47; table 1 for coefficients). Effect of temperature anomaly on the proportion of late instars increased with Bt acreage (figure 1*c*).

## 4. Discussion

We analysed how temperature anomaly and Bt adoption affects insect biology, transgenic crop–pest interactions, and agricultural pest management through transgenic biotechnology. Our results suggest that temperature anomaly and its interaction with increased Bt adoption accelerated the field-evolved resistance in *H. zea* to Bt corn expressing Cry1Ab toxin. We provide field validations of predictions from simulation models for *H. zea* [42,43], demonstrating that the elevated selection pressure from extensive adoption of Bt corn in the mid-Atlantic region has enabled *H. zea* to develop resistance to Cry1Ab expressing Bt varieties. The ‘moderate dose’ delivered by Bt crops through Cry proteins provides incomplete control [40] and increases selection pressure for resistance development. Decreasing compliance with the 20% structured refuge requirement for single Cry protein expressing hybrids [55], and the shift to refuge-in-the-bag corn hybrids that contain a blend (e.g. 95 : 5 Bt : non-Bt seed) which is less effective than structured refuge [56], also increases selection pressure. While the evolution of resistance with increased Bt adoption was expected, our study highlights the important interactive effect of climate change (temperature anomaly) for resistance development.

Extensive regional adoption of Bt corn suppressed the population abundance of *H. zea* in Maryland, similar to other reports on regional suppression of pests by Bt crops [14,17,21–23]. Although populations were reduced overall, *H. zea* abundance increased at low Bt acreage with temperature anomaly probably through increased voltinism [7,30], as they go through two to three generations in Maryland during the growing season. These interactive and/or additive influences affected the field-evolved resistance to Cry1Ab expressing Bt sweet corn. Previous anecdotal report alludes to hot weather and high Bt acreage as reasons for the failure of Bt crops (Bt cotton) against *H. zea* in Texas [57].

The mechanisms through which the interactive influences of increased Bt acreage and temperature anomaly influence evolution of *H. zea* resistance to Cry protein in Bt corn are complex as climate change impacts span spatial scales (local to global) and levels of biological organization (molecular to biosphere) [58,59]. Reduced toxicity at higher temperatures is observed for some conventional insecticides [60–62]. Also, physiological mechanisms such as increased rates of metabolic processes, excretion and the reactivity at site of action play an important role in insect resistance to conventional pesticides at warmer temperatures (e.g. *Musca domestica* L. resistance to pyrethroids; [60]). Regarding Bt crops, the activity and expression of alkaline phosphatase in the midgut binding sites is associated with resistance against Cry1F and Cry1Ac in many target lepidopteran pests including *H. zea* [63–65]. The cross-resistance between the Cry1A proteins (1Ab, 1Ac, Cry1A.105) in *H. zea* [66,67], and increased activity of alkaline phosphatase at higher temperatures [66–68] probably catalysed the field-evolved resistance to Cry1Ab particularly given high selection pressure from increased Bt adoption.

At the local spatial scale, increased feeding at high temperatures [7,8] resulted in high injury to Bt sweet corn. High temperatures may stress Bt crops and possibly degrade soluble proteins, resulting in decline of Cry1A toxin expression by the plant, and thereby reduced field efficacy [32]. Similarly, the accelerated phenology, population growth and voltinism [5,7,30] at warmer temperatures may buffer against population reduction from Bt toxin during resistance evolution. Additionally, increased overwintering survival due to warmer temperatures, particularly reduced length of time spent at near-zero temperatures [39], affect the population dynamics of target species in Bt crops and can increase the resistant allele frequency that carries over to the following spring season [68]. Concurrently, at regional/continental spatial scales, the south–north migration of moths already selected for resistance on Bt corn and cotton, that also are Bt tolerant [69], probably enhanced the development of Cry1Ab resistance in Maryland populations. The high regional deployment of Bt corn in the mid-Atlantic region, as well as further south along the migratory route of *H. zea*, probably exacerbated the selection pressure for resistance development.

Our results have important implications for understanding the risk of resistance increasing and spreading, sustainability of Bt technology and regional insect pest management strategies for climate change preparedness. The expansion of overwintering range north of 40° N, increased migration due to climate change [11,25], and the selection pressure exerted by extensive Bt acreage in mid-Atlantic, north central and northern great plains regions in the US exacerbates the risk of resistance increasing and spreading. These results highlight the necessity for adapting existing regulatory policies on refuge requirement and resistance monitoring. *Helicoverpa zea* sampling for monitoring and testing for resistance is currently concentrated in the southern US regions where both Bt corn and Bt cotton are grown. Expanded resistance monitoring for all Cry proteins registered against corn earworm in all high corn production regions would help detect resistance development as the overwintering range of corn earworm expands due to climate change.

Our study highlights risks and limitations of Bt technology for agricultural pest management in the context of climate change. Polyphagous, migratory species such as *H. zea* can benefit from warmer temperatures [7,8], and increased Bt exposure might lead to resistance development particularly in insects for which Cry proteins are moderately toxic. Hence there is a need to incorporate the evolutionary processes affected by climate change into resistance management programmes. Given the role of temperature and increased Bt adoption for resistance development, incorporating insect resistance management plans into broader integrated pest management strategies (biological control, crop rotation, etc.) could ameliorate selection pressure, and facilitate sustainability of Bt technology [24].

The evolutionary basis of *H. zea* resistance to Cry1Ab is driven by environmental conditions, and a complex genetic basis underlies insect resistance to Cry1A Bt toxins in Lepidoptera species [70]. We endeavoured to analyse the role of key environmental factors that also changed over time and may drive resistance evolution (namely Bt adoption and climatic conditions). While we highlight the statistical association of temperature anomaly and Bt acreage, we acknowledge the correlative nature of our study and the need for further research and detailed experiments. Detailed experiments relating toxicity and resistance development at varied Bt concentrations to temperature conditions are needed to clearly identify the mechanisms through which Bt adoption and climate change interactively influence field-evolved resistance.

## Supplementary Material

Dataset S1

## Data accessibility

All the relevant data used for analysis are provided as electronic supplementary material, Dataset S1.
